# Shen-Regulating Acupuncture for PTSD in Domestic Violence Survivors: A Case Series Protocol

**DOI:** 10.1192/j.eurpsy.2026.11879

**Published:** 2026-07-31

**Authors:** H.-H. Lee

**Affiliations:** Department of Traditional Chinese Medicine, Chang Gung Memorial Hospital, Taoyuan, Taiwan

## Abstract

**Introduction:**

**PTSD** is highly prevalent among **domestic violence (DV) survivors**, including women exposed to intimate partner violence (IPV), individuals with childhood DV, and socioeconomically disadvantaged persons. Approximately **26% of suicide deaths** among women in mental health care are linked to DV, highlighting the urgent need for effective interventions.

While trauma-focused therapies and SSRIs provide partial symptom relief, many patients remain symptomatic. **Acupuncture**, widely recognized for pain management, offers a **distinct Shen-regulating pathway** for emotional and somatic balance. Recent research(2023-2025) on acupuncture for PTSD shows **moderate-to-significant symptom improvements** over 4–15 weeks (two sessions per week), but evidence for DV-related PTSD is scarce and effect sizes vary.

**Objectives:**

This case series investigates **Shen-regulating acupuncture** in DV-related PTSD within an **integrative psychiatry framework**. We aim to:

1. Assess feasibility and preliminary efficacy on PTSD symptoms and emotional regulation, highlighting patient-centered outcomes.
2. Examine **mechanistic distinctions** from analgesic acupuncture, emphasizing emotional and somatic regulatory pathways.
3. Document **operational techniques** and precautions to ensure reproducibility and clinical safety.
4. Explore its role as an adjunct for **long-standing **, **treatment-resistant** trauma, offering potential benefits for patients with enduring symptomatology, including high-risk populations.

**Methods:**

1. **Participants:** DV-related PTSD patients will be recruited from the TCM department of Chang Gung Memorial Hospital, a major teaching hospital in northern Taiwan.
2. **Intervention:** 1–5 Shen-regulating acupuncture sessions, with GV20 as the core point.
3. **Outcomes:** PTSD severity (CAPS-5, PCL-C), sleep quality, emotional regulation, somatic symptoms, and heart rate variability (HRV), and other relevant measures.
4. **Study protocol:** systematically captures patient-centered clinical signals, ensuring reproducibility and safety.

**Results:**

Preliminary clinical impressions from practice suggest that, when **skillfully applied**, Shen-regulating acupuncture may elicit **noticeable improvements in emotional and somatic symptoms** as early as **one session**, with **most patients experiencing benefits within 1–3 sessions**, even for trauma persisting decades. The present protocol is designed to systematically evaluate these patient-centered clinical signals.

**Conclusions:**

Shen-regulating acupuncture may **safely complement trauma-focused psychological therapy** for DV-related PTSD, providing a **rapid, reproducible adjunct** with **mechanisms distinct from analgesic acupuncture**. It demonstrates improvements in emotional regulation and somatic symptoms, highlighting a **novel contribution to integrative psychiatry** and offering potential benefits for populations at high suicide risk.

**Disclosure of Interest:**

None Declared

